# Exploring the Relationship between Ridesharing and Public Transit Use in the United States

**DOI:** 10.3390/ijerph15081763

**Published:** 2018-08-16

**Authors:** Yuanyuan Zhang, Yuming Zhang

**Affiliations:** 1 School of Management, Shandong University, Jinan 250100, China; sduyyzhang@163.com or yzhang2@stern.nyu.edu; 2 Stern School of Business, New York University, New York, NY 10012, USA

**Keywords:** ridesharing, public transit, 2017 NHTS (National Household Travel Survey), ZINB model

## Abstract

Car travel accounts for the largest share of transportation-related greenhouse gas emissions in the United States (U.S.), leading to serious air pollution and negative health effects; approximately 76.3% of car trips are single-occupant. To reduce the negative externalities of cars, ridesharing and public transit are advocated as cost-effective and more environmentally sustainable alternatives. A better understanding of individuals’ uses of these two transport modes and their relationship is important for transport operators and policymakers; however, it is not well understood how ridesharing use is associated with public transit use. The objective of this study is to examine the relationships between the frequency and probability of ridesharing use and the frequency of public transit use in the U.S. Zero-inflated negative binomial regression models were employed to investigate the associations between these two modes, utilizing individual-level travel frequency data from the 2017 National Household Travel Survey. The survey data report the number of times the respondent had used ridesharing and public transit in the past 30 days. The results show that, generally, a one-unit increase in public transit use is significantly positively related to a 1.2% increase in the monthly frequency of ridesharing use and a 5.7% increase in the probability of ridesharing use. Additionally, the positive relationship between ridesharing and public transit use was more pronounced for people who live in areas with a high population density or in households with fewer vehicles. These findings highlight the potential for integrating public transit and ridesharing systems to provide easier multimodal transportation, promote the use of both modes, and enhance sustainable mobility, which are beneficial for the environment and public health.

## 1. Introduction

According to the United States (U.S.) Environmental Protection Agency (EPA)’s report, in 2016, the transportation sector was the largest source (28.5%) of greenhouse gas emissions in the U.S., leading to serious air pollution and negative health effects [1]. Cars accounted for the largest share (41.6%) of transportation-related greenhouse gas emissions. Americans rely highly on cars, and the 2016 American Community Survey reported that approximately 76.3% of people drive alone (single-occupant) to work, while 9.0% use ridesharing services and 5.1% use public transit [2]. Single-occupant trips combined with the increasing number of cars on the road lead to severe congestion, more vehicle emissions, increased fuel use, and stress among people.

To reduce the negative externalities of cars, ridesharing and public transit are advocated as cost-effective and more environmentally sustainable alternative transportation modes [3,4]. Ridesharing refers to mobile-enabled on-demand mobility services provided by rideshare platforms (e.g., Uber, Lyft, and Didi) [5]. Some studies have investigated the environmental benefits of ridesharing services, such as greenhouse gas emission reductions, decline in the traffic congestion, and fuel savings [6,7,8,9]. Ridesharing enables individuals to maintain convenience, flexibility, and a degree of luxury by relying on cars, and ridesharing is also cost-effective in many cases [10]. Public transit systems cost less but are always fixed-line [11]. 

To attract more riders to use ridesharing and public transit, some local government agencies have subsidized passengers’ use of ridesharing services to accommodate the first and last mile of public transit and to better coordinate mobility in the U.S. [12]. The integration of ridesharing and public transit systems is proven to significantly enhance mobility, and a detour-based pricing mechanism for the connection of these two modes is designed to improve the use of rail public transit [13].

A systematic understanding of how these two transport modes relate to each other is important for transportation agencies and governments. Previous studies have found that the associations between ridesharing and public transit use may be complementary or substitutive [14,15,16,17]. However, how ridesharing use is associated with public transit use is not well understood. This study aims to examine the relationships between ridesharing and public transit use in the U.S., utilizing individual-level frequency data from the 2017 National Household Travel Survey (NHTS). Zero-inflated negative binomial (ZINB) regression models were constructed to examine the associations, and the results show that public transit use is positively related to ridesharing use. The positive relationship between ridesharing and public transit use was more pronounced for people who live in areas with high population density or in households with fewer vehicles. These findings highlight the potential for integrating public transit and ridesharing systems to provide easier multimodal transportation, promote the use of both modes, and enhance sustainable mobility.

This study has two main contributions. First, we provide empirical evidence of how and to what extent the individual’s ridesharing use is related to public transit use and how the relationships vary across different regions and households. The findings offer important implications for governments and transit operators to decide the degree to which they subsidize or cooperate with ridesharing service providers, or where it is beneficial to adjust the supply of public transit services. Second, previous studies used agency-level data [15] or data from a single city [17], but they have not considered the actual frequency of ridesharing and public transit use at the individual level. To our knowledge, this is the first study to quantify the relationships between these two modes, and to use individual-level frequency of travel data from a nationwide travel survey. From the methodological perspective, we employed ZINB models to analyze the frequency data. 

The remainder of the study is organized as follows. Related studies on the associations between ridesharing and public transit are described in Section 2. In Section 3, the data used, and descriptive analysis are presented. Section 4 presents the methodology. The results are presented in Section 5, and Section 6 provides some discussion. Finally, Section 7 concludes this study.

## 2. Literature Review

For the ridesharing research fields, previous studies have discussed the classification of ridesharing systems [10], ride-matching algorithms for ridesharing systems [18,19], dynamic ridesharing pricing [20,21], trust among peers [22], privacy protection problems [23], socio-economic impacts of ridesharing services [24], and environmental effects of ridesharing [6,9,25,26].

An individual’s transportation mode choice is influenced by a set of factors, such as travel cost, travel distance, travel time, convenience, vehicle ownership, socio-demographics, built environments, cultures, personal attitudes, and perceptions of safety [27,28,29,30]. Some studies have examined the factors influencing the use of ridesharing services, which include perceptions of availability and safety [3], travel cost and time of travel [31], gasoline prices [32], and some demographic variables (e.g., age, education level, and income level) [33,34]. 

Only a limited number of prior studies are related to our research question. The associations between ridesharing and public transit may be complementary or substitutive, and conclusions from prior studies on this research question are mixed. At present, how ridesharing is related to public transit is not well understood. The existing studies on the relationships between ridesharing and public transit use are summarized as follows. 

Rayle et al. [17] examined how ridesharing complements or competes with public transit using survey data with 380 respondents in San Francisco and found that ridesharing appears to both a substitute for and complement to public transit; ridesharing seems to substitute for public transit for some individual trips, but for the majority of the trips, ridesharing complements public transit. Approximately one-third of respondents reported that they often chose to use ridesharing services rather than public transit due to its travel time savings. However, the generalizability of their study is questionable because the survey sample is small and focuses on a single city; therefore, we used national-level survey data to analyze the associations between individuals’ ridesharing and public transit use. 

Babar and Burtch [15] evaluated the effects of ridesharing service entry on the use of public transit over the subsequent 12 months by constructing a difference-in-difference model using agency-level data. They indicated that Uber substituted for road-based short-distance public transit trips, which is evidenced by a 1.05% decrease in the use of city buses over the subsequent 12 months following Uber’s entry. They also found that Uber complemented rail-based long-distance public transit trips; Uber’s entry was related to a 2.59% increase in the use of subways and a 7.24% increase in the use of commuter rails over the subsequent 12 months. However, their study examined the effects of ridesharing service entry on the use of public transit at the agency level and did not consider the individual’s actual ridesharing use (the frequency and probability of ridesharing use) at the individual level.

Stiglic et al. [16] conducted a computational study to investigate the potential benefits of integrating ridesharing and public transit. They found that the integration of ridesharing and public transit systems can potentially increase the use of public transit, and the matching rate increases from 66.8% in a single ridesharing system to 83.8% in an integrated system. Bian and Liu [13] designed a detour-based discounting mechanism for those who use ridesharing as a first-mile choice to a public transit station. Ridesharing seems to be more economical and convenient to address the first- and last-mile problems for those who drive and park or are dropped off by others at stations, sparing them worry about parking near the station or reliance on friends or families for a ride to a station, and this complementary situation is more common for work or school commuters [5]. Murray [14] reported that ridesharing was working as a complement to public transit to address the first- and last-mile problems.

Overall, the existing empirical evidence of the associations between ridesharing and public transit use is mixed. The conflicting conclusions of previous studies may be due to differences in empirical methods or different data sources. Our study adds further evidence to this issue by utilizing individual-level travel frequency data from a national household travel survey. We conducted descriptive statistics using graphs in Section 3, to intuitively present the relationships between ridesharing and public transit use; then, the ZINB models were employed to further examine the associations between these two transport modes, and the empirical analysis results were reported in Section 5.

## 3. Data

### 3.1. Data Source

The 2017 NHTS was conducted by the U.S. Department of Transportation administration from March 2016 to May 2017 [35], with the aim to better understand travel behaviors of the U.S. population. The 2017 NHTS was a randomized, voluntary, large-scale national travel survey. The first phase of the survey was the household recruitment survey, from which the household respondents were recruited by address-based random sampling with mail-back technology, and household socioeconomic and geographic characteristics were collected; the weighted response rate of this phase was 30.4%. The second phase of the survey was the person-level retrieval survey, which gathered information about the respondents’ (all the individuals in the households that were recruited) detailed travel behaviors and demographics using a phone- or web-based response mode; the weighted response rate of this phase was 51.4%. The overall weighted survey response rate was 15.6%, which included 264,234 individuals and 129,696 households. 

The number of times ridesharing was used in the past 30 days was defined using the survey question, “how many times have you purchased a ridesharing service with a smartphone rideshare application (e.g., Uber, Lyft, or Sidecar) in the past 30 days?” A total of 236,089 individuals answered this question about ridesharing use. The frequency of public transit use in the past 30 days was defined using the survey question, “how many times have you used public transportation (e.g., buses, subways, or commuter trains) in the past 30 days?” A total of 206 individuals were excluded from the 236,089 observations because of missing data on this public transit use question. We eliminated 9265 observations because of missing information on some important characteristics (e.g., gender, age, education level, race, household income level, household vehicle ownership, and population density at the home location). The sample retained and used in this study includes 226,824 individuals. The software STATA 13.1 (College Station, TX, USA) was used to perform all statistical analyses in this study.

### 3.2. Descriptive Analysis of Ridesharing Use

**Figure 1:**
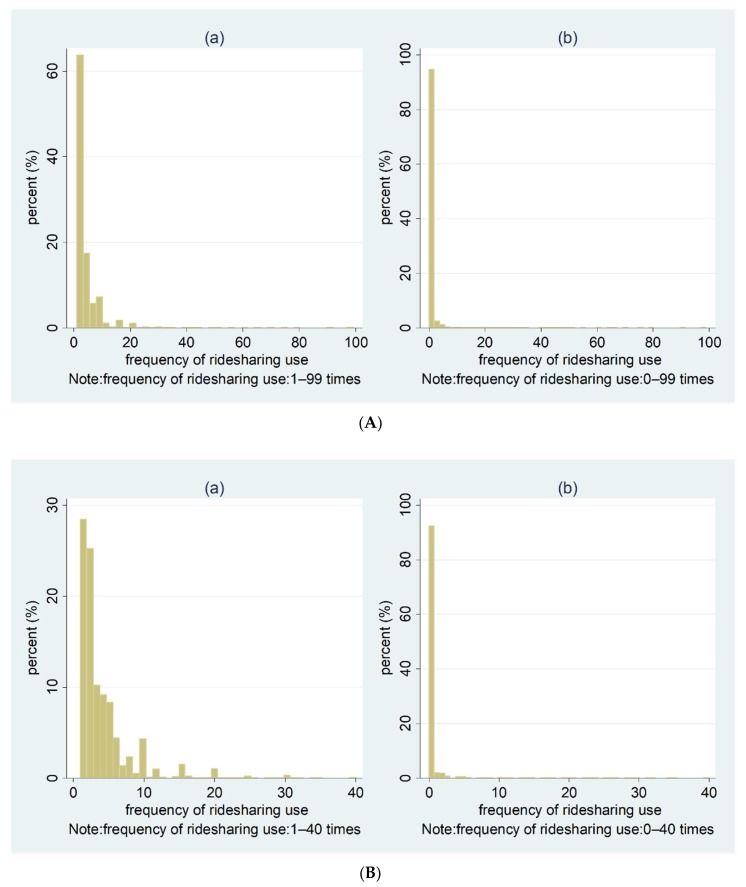
(**A**) Distribution of the frequency of monthly ridesharing use for those who used ridesharing in the past 30 days: (**a**) 1–99 times; (**b**) 0–99 times. (**B**) Distribution of the frequency of monthly ridesharing use for those who used ridesharing in the past 30 days: (**a**) 1–40 times; (**b**) 0–40 times.

Individuals were asked to provide the number of times (frequency) they had used ridesharing in the past 30 days, and the number ranged from zero to 99 times. Figure 1A shows the distribution of the frequency of ridesharing use. In all, 209,794 (92.49%) people reported that they did not purchase a ridesharing service at all in the past 30 days, while 17,030 (7.51%) individuals reported that they had used a ridesharing service at least once (1–99 times) in the past 30 days. Among those who had used a ridesharing service 1–99 times in the past 30 days, 4835 (28.39%) people had used ridesharing once, 4287 (25.17%) had used ridesharing twice, and 13,840 (81.27%) had used ridesharing less than five times; 2215 (13.01%) had used ridesharing 6–10 times; 771 (4.53%) had used ridesharing 11–20 times; 165 (0.97%) had used it 21–40 times; and only 39 (0.23%) had used ridesharing 41–99 times in the past 30 days. To better tick the values on the x-axes, we used Figure 1B to show the distribution of the frequency of ridesharing use for those who used ridesharing (a) 1–40 times and (b) 0–40 times.

The dependent variable is the number of times ridesharing had been used in the past 30 days, which is the count outcome, and the fittest count modeling technology for this study is the ZINB model (the reason why the ZINB model is the best model for the analysis will be explained later in the methodology section). The *p*-value < 0.05 was considered to be statistically significant. The sample size for this study was 226,824, with 209,794 zero values and 17,030 non-zero values for the frequency of ridesharing use in the past 30 days. A binomial test with unequal sample sizes (ratio = 0.0751/0.9249, the proportion of zero values/the proportion of non-zero values) was employed to compute the statistical power. For this study, the significance level was 0.05, and the sample size was 226,824; in this case, the calculated statistical power was 1. Therefore, the sample size of 226,824 was large enough to provide robust statistical power.

**Figure 2:**
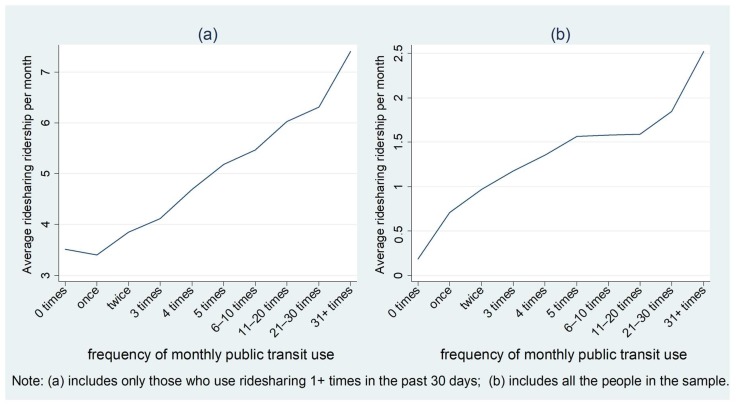
Average monthly ridesharing use varying by the frequency of public transit use. (**a**) includes only those who use ridesharing 1 + times in the past 30 days; (**b**) includes all the people in the sample.

**Figure 3:**
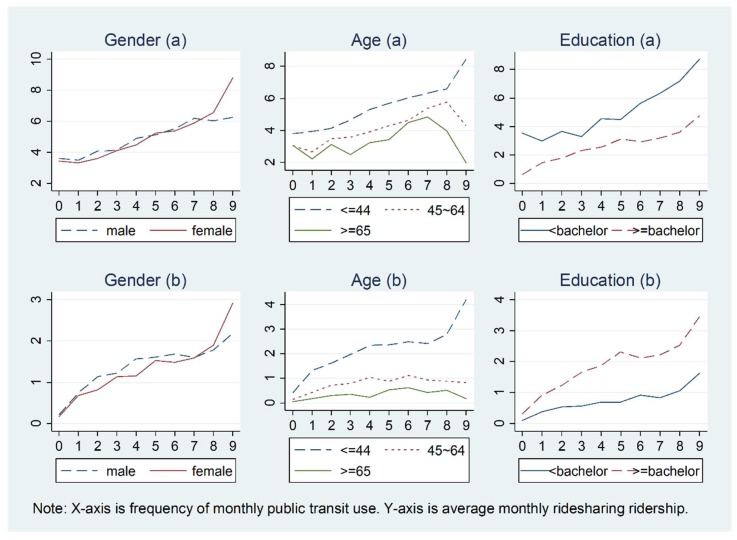
Relationship between ridesharing and public transit use varying by gender, age, and education level for those who use ridesharing in the past 30 days: (**a**) 1–99 times; (**b**) 0–99 times. (for X-axis, 0 = 0 times, 1 = once, 2 = twice, 3 = 3 times, 4 = 4 times, 5 = 5 times, 6 = 6–10 times, 7 = 11–20 times, 8 = 21–30 times, and 9 = 31–99 times; the same for X-axis in Figure 4, Figure 5, Figure 6 and Figure 7).

**Figure 4:**
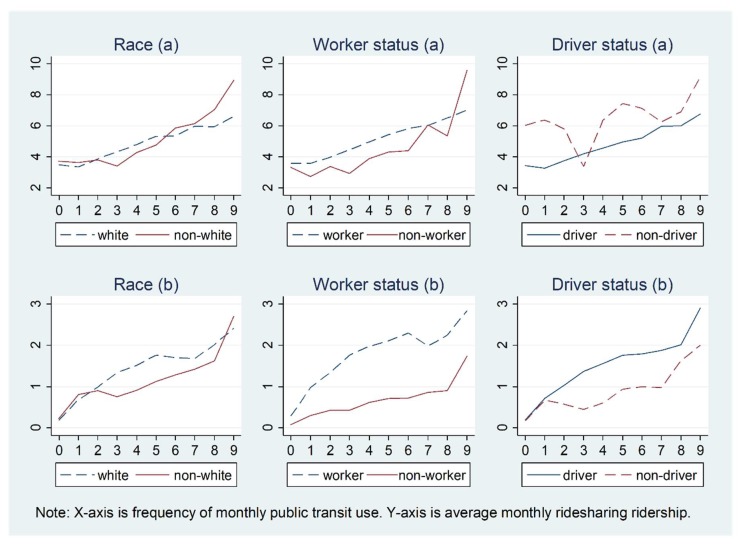
Relationship between ridesharing and public transit use varying by race, worker, and driver status for those who use ridesharing in the past 30 days: (**a**) 1–99 times; (**b**) 0–99 times.

**Figure 5:**
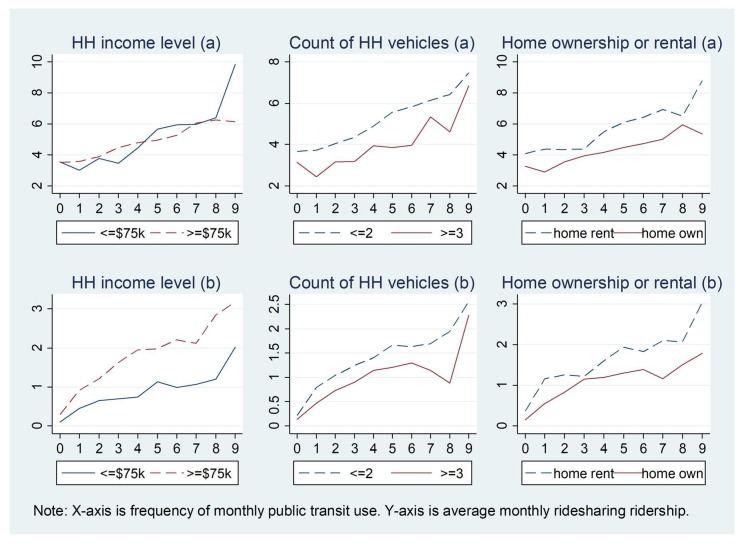
Relationship between ridesharing and public transit use varying by household characteristics for those who use ridesharing in the past 30 days: (**a**) 1–99 times; (**b**) 0–99 times.

**Figure 6:**
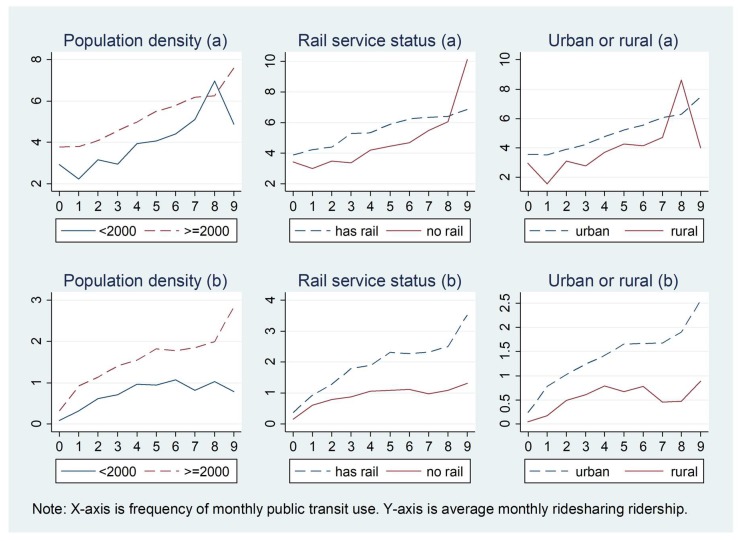
Relationship between ridesharing and public transit use varying by geographic characteristics for those who use ridesharing in the past 30 days: (**a**) 1–99 times; (**b**) 0–99 times.

**Figure 7:**
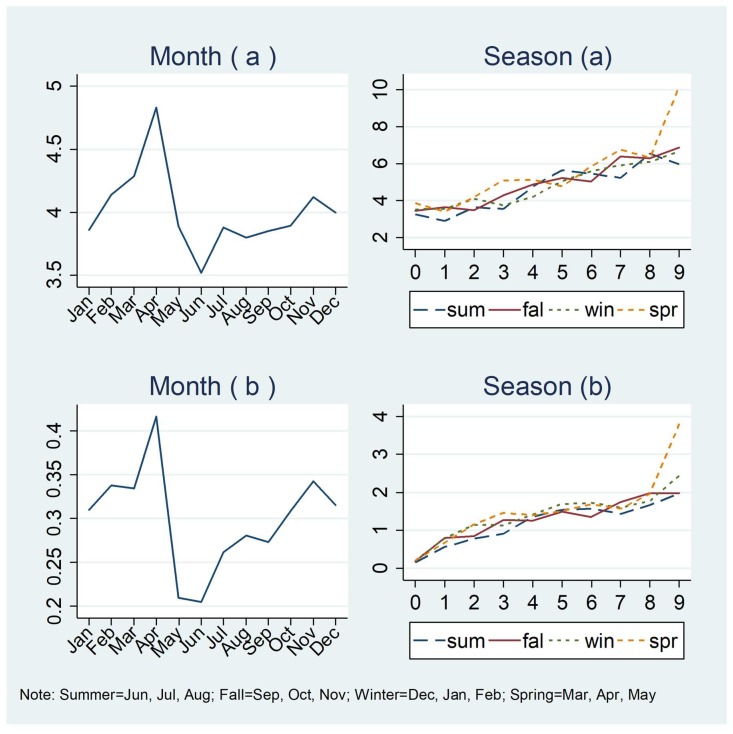
Average monthly ridesharing use varying by month and season for those who use ridesharing in the past 30 days: (**a**) 1–99 times; (**b**) 0–99 times.

Figure 2 shows how the average ridesharing use per month varies by the frequency of public transit use in the past 30 days. For Figure 2, Figure 3, Figure 4, Figure 5, Figure 6 and Figure 7, panels (a) include people who used the ridesharing service at least once in the past 30 days, and panels (b) include all the individuals in the sample. From Figure 2, we can see that the frequency of public transit use was positively related to the average monthly ridesharing use in the past 30 days. For those who used the ridesharing service more than once, the average ridesharing use per month was 4.01 rides, while the number was 0.30 for the whole sample.

Figure 3 and Figure 4 show how the relationship between ridesharing and public transit use in the past 30 days varies by personal demographics. For Figure 2, Figure 3, Figure 4, Figure 5 and Figure 6 and Figure 7b, the X-axis is the number of times public transit was used (frequency): 0 denotes 0 times, 1 denotes once, 2 denote twice, 3 denotes three times, 4 denotes four times, 5 denotes five times, 6 denotes six to ten times, 7 denotes 11–20 times, 8 denotes 21–30 times, and 9 denotes more than 31 times. In general, a very similar pattern for the relationships between ridesharing and public transit use are observed for men and women, white and non-white individuals, and workers and non-workers, while the associations are inconsistent for younger people and older people, those with high and low education levels, and drivers and non-drivers. On average, younger people use ridesharing more frequently than older people, and ridesharing use is clearly positively related to public transit use only for the age group of less than 44 years old. Workers use ridesharing more frequently than do non-workers (the average number of times ridesharing is used by workers and by non-workers is 0.45 and 0.12, respectively, for all the people in the sample, and the number is 4.10 and 3.64, respectively for those who used ridesharing at least once in the past 30 days).

Figure 5 shows how the relationships between ridesharing and public transit use in the past 30 days vary by different household income levels, household vehicle ownership, and home ownership. The associations between ridesharing and public transit use show very similar patterns for different household income level, count of household vehicles, and home ownership groups, from which we can see that ridesharing use is positively associated with public transit ridership for all three different groups. Individuals in households with fewer vehicles use ridesharing more frequently than those in households with more vehicles. Individuals living in rental houses show a higher frequency of ridesharing use than those who own their houses.

Figure 6 shows how the associations between ridesharing and public transit use in the past 30 days vary by geographic characteristics at the home location, including population density, rail service status, and urban status. The relationships between ridesharing and public transit use are similar for these three different groups, suggesting that ridesharing use is positively related to public transit use within all the groups. Individuals living in areas with higher population density use ridesharing more frequently than those who live in areas with lower population density. People who live in areas without rail service show a lower frequency of ridesharing use than those living in areas with rail service.

Figure 7a shows how the average monthly ridesharing use varies by month, and Figure 7b shows how the relationship between ridesharing and public transit use varies by season. The average number of times ridesharing is used per month shows the highest frequency of ridesharing use in the spring months, while the lowest frequency of ridesharing use is in the summer months. The relationships between ridesharing and public transit use show almost no difference for different seasons, indicating that ridesharing ridership has a positive relationship with public transit use in the past 30 days for all seasons.

### 3.3. Variable Definitions and Descriptive Statistics

The dependent variable, Rideshare, is measured by the number of times (frequency) an individual used ridesharing in the past 30 days. The range of Rideshare is from 0 to 99, and the mean number of ridesharing use is 0.30. The independent variable, Ptused, is measured by the number of times (frequency) public transit was used in the past 30 days. The average monthly frequency of public transit use is 0.88 for the whole sample, and the value of this variable ranges from 0 to 240. Table 1 provides the definitions and descriptive statistics for all the variables.

Some other variables may influence individuals’ ridesharing use, so we controlled for four groups of variables in this study, including personal demographics, household socioeconomic characteristics, geographic characteristics at the home location, and seasons. First, personal demographics were measured by whether the individual was female (Female), the individual’s age in years (Age), the individual’s education level (Education), whether the individual’s ethnicity was white (White), whether the individual was a worker (Worker), and whether the individual was able to drive (Driver). Second, household socioeconomic characteristics were measured by the individual’s household income level (HHincome), the number of vehicles in the household (HHvehcount), and whether the house was a rental (Homerent). Third, geographic characteristics were measured by population density (persons per square mile) at the home location (Pdensity), whether the area where the house was located has rail service (Rail), and whether the house was in an urban area (Urban). Lastly, seasons were measured by four dummy variables, including whether the survey was conducted in March, April, or May (Spring), whether the survey was conducted in June, July, or August (Summer), whether the survey was conducted in September, October, or November (Fall), and whether the survey was conducted in December, January, or February (Winter).

## 4. Methodology

In this section, at first, we discussed the reason for choosing zero-inflated negative binomial (ZINB) regression models as the methodological approaches to analyze the data. Then, the distribution and function of the ZINB model were presented. Finally, we presented how to apply the ZINB models in this study.

### 4.1. Model Selection

The dependent variable of this study was the number of times ridesharing was used in the past 30 days, which was a discrete non-negative count outcome. Regarding methodological approaches for count outcome, Poisson regression models and negative binomial (NB) regression models are often used to address the “count” characteristics of frequency data [36]. The Poisson models require that the mean and variance of the variable be approximately equal; however, the Poisson models cannot account for the problem of over-dispersion (the mean is obviously smaller than the variance of the data) [37]. The over-dispersion problem may result in biased, inefficient parameter estimates [33]. NB models relax the limitation that the mean and variance must be equal and are more appropriate for handling the over-dispersion problem of the data [38].

When the data have a large number of zero counts for the dependent variable, this may lead to the probability of zero-inflated problems; however, Poisson models and NB models cannot handle the zero-inflated problems [37]. The zero-inflated models (extended forms of traditional Poisson and NB models) include zero-inflated Poisson (ZIP) regression models and zero-inflated negative binomial (ZINB) regression models and can address the possibility of excess zero counts for the predicted variable [37,39]. The ZIP models have the constraint that the variance must be equal to the mean of the variable, while the ZINB models can handle over-dispersion problems [40]. 

A number of studies have used ZINB models to analyze data that are over-dispersed and zero-inflated. A literature review of studies on transport safety themes indicated that zero-inflated models are more suitable to be used as modeling approaches when the zero counts of the observed data are over 65% [41]. Shen and Neyens [42] employed ZINB models to study the relationships between the length of hospital stay of teen drivers and possible crash-related factors, and the zero values of the dependent variable for girl and boy drivers were 96.7% and 94.2%, respectively. For this study, the data of the dependent variable were both over-dispersed (the variance 2.30 was greater than the mean 0.30) and zero-inflated (zero values account for 92.49% of the observations); therefore, ZINB models were the best modeling techniques to conduct the statistical analyses.

### 4.2. Zero-Inflated Negative Binomial Regression Model

#### 4.2.1. Distribution of the ZINB Model

ZINB models have two distinct count data generating processes [40,43]. The first counting process is the true zero-count process (zero state, odds of always being 0), which is expressed as a logit model with the probability of $p_{i}$; the second counting process is the count-data process (non-zero state, odds of not always being 0), which is expressed as an NB model with the probability of ($1-p_{i}$). Zero values are generated from both of these counting processes; therefore, the overall probability of zero counts is the combination of the probability of zeros from these two processes. Let *y* denote the number of times ridesharing was used in the past 30 days; let P(y=0) and P(Y=y) denote the probability of zero count and non-zero counts, respectively. Therefore, the distribution of the ZINB model could be written as follows:

(1) $$P(y=0)=p_{i}+(1-p_{i}){\left(\frac{1/t}{\theta_{i}+1/t}\right)}^{1/t}\;$$

(2) $$P(Y=y)=(1-p_{i})\frac{\Gamma (y+1/t)}{\Gamma (1/t)\Gamma (y+1)}{\left(\frac{1/t}{\theta_{i}+1/t}\right)}^{1/t}{\left(\frac{\theta_{i}}{\theta_{i}+1/t}\right)}^{y}y=1,2,3,\ldots \;$$

where *t* denotes the dispersion parameter of the second counting process (NB model) and $\theta_{i}$ denotes the mean of the variable. The mean and variance of the dependent variable are expressed as follows:

(3) $$E(Y)=(1-p_{i})\theta_{i}\;$$

(4) $$Var(Y)=(1-p_{i})\left(1+\theta_{i}t+p_{i}\theta_{i}\right)\theta_{i}.$$

#### 4.2.2. ZINB Mixed Model

Let $y_{ij}(i=1,2,\ldots m;j=1,2,\ldots n_{i})$ denote a count that is the *j*th observation of the *i*th cluster; the total number of clusters and observations are *m* and $\sum_{i=1}^{m}n_{i}=n$, respectively. For the ZINB model, $\log(\frac{p_{ij}}{1-p_{ij}})$ is the logit component and $\log(\theta_{ij})$ is the NB component, and both of these components are assumed to depend on linear functions of a set of covariates (explanatory variables). The ZINB regression model can be written as follows:

(5) $$\log(\frac{p_{ij}}{1-p_{ij}})=\varphi_{ij}=V_{ij}{}^{{}^{T}}\beta +\psi_{i}\;$$

(6) $$\log(\theta_{ij})=\xi_{ij}=W_{ij}{}^{{}^{T}}\gamma +\eta_{i}\;$$

where $\varphi_{ij}$ and $\xi_{ij}$ are predictors of the two components; $V_{ij}$ and $W_{ij}$ are explanatory variables of these two components, respectively, and $V_{ij}$ and $W_{ij}$ are not necessarily the same; β are vectors of coefficients of the logit component, and γ are vectors of coefficients of the NB component. Let the vectors $\psi_{i}={\left(\psi_{1},\ldots ,\psi_{m}\right)}^{T}$ and $\eta_{i}={\left(\eta_{1},\ldots ,\eta_{m}\right)}^{T}$ be the cluster-level random variations of these two components, respectively; $\psi_{i}$ are assumed to be independent as $N(0,\sigma_{\psi }^{2}U_{m})$ and $\eta_{i}$ are assumed to be independent as $N(0,\sigma_{\eta }^{2}U_{m})$, where $U_{m}$ is an m×m matrix. Maximum likelihood methods were used to estimate the coefficients appearing in the ZINB models.

#### 4.2.3. ZINB Model Application

In this study, ZINB models were used to discuss the relationships between ridesharing and public transit use. The vectors of $V_{ij}$ and $W_{ij}$ are the same covariates for our analysis models. The non-zero state (NB component) was used to examine the associations between the frequency of ridesharing use and public transit use in the past 30 days; the zero state (logit component) was employed to examine the associations between the probability of ridesharing use and public transit use in the past 30 days.

## 5. Results

The detailed results of the ZINB models are presented in this section. We examined the associations between the frequency of ridesharing use (the number of times ridesharing was used in the past 30 days) and the frequency of public transit use, and the result was shown in the non-zero state; we also investigated the associations between the probability of ridesharing use (whether ridesharing was used at least once or never in the past 30 days) and the frequency of public transit use, and the result was shown in the zero state. Generally, public transit use is significantly positively related to ridesharing use, indicating that the increase in the frequency of public transit use is positively associated with the increase in the frequency and probability of ridesharing use. The relationships between ridesharing and public transit use are affected by population density at the home location, so we employed ZINB models to examine how the relationship between ridesharing and public transit use varies by population density. The number of vehicles in the household also influences the associations between ridesharing and public transit use, so we constructed ZINB models to examine the association between ridesharing and public transit use varied by the household vehicle ownership.

### 5.1. Results for the Relationship between Ridesharing and Public Transit Use

Table 2 presents the results for the ZINB model. The marginal effects ($e^{\gamma }-1$) in the non-zero state denote the percent change in the frequency of ridesharing use for a one-unit increase in an explanatory variable after controlling for the other variables. To be more specific, a one-unit increase in public transit use in the past 30 days is positively associated with a 1.2% increase in the frequency of ridesharing use, with the significance level of 0.1% (*p*-value < 0.001). Let SD be the standard deviation of the independent variable and the marginal effects ($e^{\gamma *SD}-1$) in the non-zero state denote the percent change in the frequency of ridesharing use for one SD increase in an explanatory variable, holding all the other variables constant. One SD increase in public transit use in the past 30 days (the SD of the variable Ptused is 4.30 for the whole sample) is positively associated with a 5.4% increase in the frequency of ridesharing use, and the result is significant at the 0.1% level (*p*-value < 0.001). 

The marginal effects ($e^{\beta }-1$) in the zero state denote the percent change in the probability of ridesharing use for a one-unit increase in an explanatory variable, holding all the other variables constant. The positive marginal effects in the zero state suggest that people are more likely to have zero values of ridesharing use and thus are less likely to use ridesharing at least once, indicating a lower probability of ridesharing use in the past 30 days. A one-unit increase in public transit use in the past 30 days is positively related to a 5.7% increase in the odds of ridesharing use, and the result is significant at the 0.1% level (*p*-value < 0.001). The marginal effects ($e^{\beta *SD}-1$) in the zero state give the percent change in the probability of ridesharing use for a one SD increase in an independent variable after controlling for all the other variables. A one SD increase in public transit use is positively related to a 22.4% increase in the probability of ridesharing use with the significance level of 0.1% (*p*-value < 0.001). We did not report the marginal effects of one SD increase in the explanatory variable, and we only reported the marginal effects for a one-unit increase in the independent variable in the results.

Table 2 also shows that the respondents’ ridesharing use is affected by control variables. Men use ridesharing 9.3% more frequently than women, but women are 11.9% more likely to use ridesharing than men. A one-unit increase in age is associated with a 1.0% and 4.1% increase in the frequency and probability of ridesharing use, respectively, suggesting that younger individuals have a higher frequency and a higher probability of ridesharing use than older people; this may be explained by the higher willingness of younger people to adopt new technology and services [33]. An increase of one education level is related to a 3.4% decrease in the frequency of ridesharing and a 37.1% increase in the odds of ridesharing use. The race and worker status show no significant effects on an individual’s frequency of ridesharing use; however, white people are 13.5% more likely to use ridesharing than those whose race is not white, and workers are 30.3% more likely to use ridesharing than are non-workers. 

People who are able to drive use ridesharing 36.0% less frequently but are 29.8% more likely to use ridesharing than those who are unable to drive. A one-level increase in household annual income is related to a 6.0% increase in the frequency of ridesharing use and a 21.7% increase in the odds of ridesharing use, suggesting that people in households with higher income levels use ridesharing more frequently and are more likely to use ridesharing. One additional vehicle in the household is associated with a 7.9% decrease in the frequency of ridesharing use and a 23.4% decrease in the probability of ridesharing use. People living in areas with a higher population density show a 14.9% higher frequency and a 23.2% higher probability of ridesharing use than those living in areas with a lower population density. Individuals living in areas with rail service use ridesharing 11.6% more frequently and are 27.1% more likely to use ridesharing than those living in areas without rail service. Compared to traveling in the fall, those who travel in the spring use ridesharing 14.7% more frequently, and those who travel in the summer use ridesharing 8.2% less frequently; this may be explained by the lower willingness of people to use ridesharing in uncomfortably hot weather.

### 5.2. Results Varying by Population Density

Table 3 shows how the relationship between ridesharing and public transit use varies by population density. The sample was divided into a high population density group (more than 2000 people per square mile at the home location) and a low population density group (fewer than 2000 people per square mile at the home location). A one-unit increase in public transit use is related to a 1.1% increase in the frequency of ridesharing use for the high population density group with a significance level of 0.1% (*p*-value < 0.001), while a one-unit increase in public transit use is associated with a 0.9% higher frequency of ridesharing use for the low population density group with a significance level of 5% (*p*-value < 0.05), suggesting that the positive associations between ridesharing and public transit use are more pronounced for those who live in areas with high population density. The positive associations between the probability of ridesharing use and the frequency of public transit use are significant at the 0.1% level (*p*-value < 0.001) for both groups.

### 5.3. Results Varying by the Number of Vehicles in the Household

Table 4 shows how the relationship between ridesharing and public transit use varies by the number of vehicles in the household. The household vehicle ownership was divided into two groups: a low number of household vehicles (fewer than two vehicles in the household), and a high number of household vehicles (more than three vehicles in the household). A one-unit increase in public transit use is associated with a 1.3% increase in the frequency of ridesharing use for the low household vehicle ownership group with a significance level of 0.1% (*p*-value < 0.001), while the positive relationship for the high household vehicle ownership group is not significant, indicating that the positive associations between ridesharing and public transit use are more pronounced for those who live in households with fewer vehicles. The probability of ridesharing use is positively related to the frequency of public transit use with the significance level of 0.1% (*p*-value < 0.001) for both groups.

## 6. Discussion

We employed ZINB models to examine the relationships between ridesharing and public transit use utilizing data from the 2017 NHTS. The results show that an individual’s public transit use is significantly positively related to the frequency and probability of ridesharing use, suggesting that an increase in the use of public transit is associated with an increase in ridesharing use. Generally, a one-unit increase in public transit use is significantly positively related to a 1.2% increase in the monthly frequency of ridesharing use and a 5.7% increase in the probability of ridesharing use, which means that people who use public transit more frequently use ridesharing more frequently and are more likely to use it than those who use ridesharing less frequently. The positive associations between ridesharing and public transit use were also discussed by Babar and Burtch [15]. However, their study did not consider the individual’s actual ridesharing use at the individual level. Our findings suggest that public transportation agencies perhaps should view ridesharing systems as opportunities rather than threats. The integration of the ridesharing and public transit systems and associated benefits have been discussed by some previous studies, which have found that such integration could improve the overall efficiency of the transportation systems [16,44].

The positive relationship between ridesharing and public transit use was more pronounced for people who live in areas with a high population density, which is evidenced by the fact that the effect of an increase in the frequency of public transit use on the increase in ridesharing use for people in more densely populated areas is greater, and the results are more significant than those in less densely populated areas. In more densely urbanized areas, people are more likely to have easier access to ridesharing services, as the on-demand mobility service market is more active and has a higher matching rate in real time, with more drivers providing ridesharing services and more riders using ridesharing services on ridesharing platforms or systems (e.g., Uber and Lyft). In addition, there are more advanced public transportation infrastructures with more system participants in denser urban areas [34,45], providing greater opportunity for travelers to combine ridesharing and public transport systems. 

Strategies of improving the quality and quantity of public transit services in more densely populated areas should be developed to retain current users and attract new ones [46,47], which is also beneficial for the increase in ridesharing service demand. The public transit operators can choose to cooperate with ridesharing service providers to offer additional benefits (such as toll waivers, HOV lane permits, and parking priorities in more densely populated areas) to drivers who are willing to accommodate riders to public transit stations, which may help increase the use of public transit and decrease the number of single-occupant vehicles on the road.

There is also a difference in the association between ridesharing and public transit use by household vehicle ownership: the positive relationship between these two transport modes is significant at the 0.1% level for people who live in households with fewer vehicles, while the association is not significant for those whose households have more vehicles. This may be explained by the reasoning that if there are more vehicles in the household, individuals have the alternative of driving a car rather than using ridesharing or public transit systems [48]. Table 2 shows that the number of vehicles in the household is significantly negatively associated with the individual’s frequency and probability of ridesharing use, and Dias et al. [33] also found that the number of vehicles in the household was negatively related to the individual’s probability of ridesharing use.

The results also indicate that ridesharing use differs across person-level demographics and household-level socioeconomic and geographic characteristics. Increased frequency and probability of ridesharing use were associated with younger age, higher household income level, lower number of vehicles in the household, higher population density, and rail service. Dias et al. [33] and Efthymiou et al. [31] found similar results in their study. These relationships could inform policy decisions targeting increased ridesharing use. Individuals’ heterogeneous ridesharing use varying by personal, household, and regional characteristics should be considered when policy makers and service providers make plans to improve ridesharing use. Therefore, companies can optimally position ridesharing services, and authorities can make appropriate incentive policies aiming to increase the use of ridesharing in a cost-effective way [46].

## 7. Conclusions

Car travel (approximately 76.3% of car trips are single-occupant) accounted for the largest share of transportation-related greenhouse gas emissions in the US, leading to serious air pollution and negative health effects. Ridesharing and public transit are advocated as cost-effective and more environmentally sustainable alternatives to reduce the above negative externalities of cars. Previous studies stated that ridesharing was related to public transit use; however, the associations between ridesharing and public transit use remain unclear. In this study, we employed ZINB models to examine the relationships between ridesharing and public transit use using data from the 2017 NHTS.

The results show that, generally, a one-unit increase in public transit use is significantly positively related to a 1.2% increase in the monthly frequency of ridesharing use and a 5.7% increase in the probability of ridesharing use, indicating that ridesharing use is positively associated with public transit use. The findings suggest that interventions and policies aiming to increase the use of ridesharing or public transit would improve the use of both of these transport modes. In addition, the positive relationship between ridesharing and public transit use was more pronounced for people who live in areas with a high population density or in households with fewer vehicles. The heterogeneous associations between these two modes across different populated areas and household vehicle ownership should be considered when interventions and policies are made. People who are young, in households with high income levels and a low number of vehicles, and in areas with high population density or rail service use ridesharing more frequently and are more likely to use ridesharing. Interventions targeting the increase in the use of ridesharing should consider the heterogeneous effects of personal, household, and geographic characteristics. The findings have implications for governments and public transit operators to decide where to subsidize or cooperate with ridesharing service providers and where to adjust the supply of public transit services.

This study has several weaknesses. First, the analysis based on cross-sectional data can be used to provide evidence of the relationships between different variables but not to infer causality. Second, while other factors (personal habits, attitudes, or culture) may influence individual’s transport mode choice [28,29] and the use of ridesharing, we could not control for such factors, as the 2017 NHTS data do not collect this information. Third, the survey question about the frequency of public transit use is “how many times have you used public transportation (e.g., buses, subways, or commuter trains) in the past 30 days?” Therefore, we cannot separate the specific effects of each public transit mode due to the missing frequency data for each mode in the 2017 NHTS data. Finally, the dependent variable of this study is frequency data (the number of times respondents used ridesharing in the past 30 days); therefore, the models can not reflect some of the ridesharing trip characteristics (e.g., trip purpose, travel time, and trip distance for each trip), and this should be addressed in future research.

## Figures and Tables

**Table 1:** Variable definitions and descriptive statistics.

Variable	Definition	Type	Obs.	Mean	Std. Dev.	Min	Max
Dependent variable							
Rideshare	Frequency of ridesharing use in the past 30 days	Ordinal	226,824	0.301	1.732	0	99
Independent variable							
Ptused	Frequency of public transit use in the past 30 days	Ordinal	226,824	0.879	4.297	0	240
Control variables							
Personal demographics							
Female	If respondent is female	Dummy	226,824	0.531	0.499	0	1
Age	Respondent’s age (years)	Ordinal	226,824	53.022	18.256	16	92
Education	Respondent’s education level: 1 = less than high school, 2 = high school/General Educational Development (GED), 3 = some college/associate, 4 = bachelor, 5 = graduate/professional	Ordinal	226,824	3.330	1.185	1	5
White	If respondent’s race is white	Dummy	226,824	0.825	0.380	0	1
Worker	If respondent is a worker	Dummy	226,824	0.549	0.498	0	1
Driver	If respondent is a driver	Dummy	226,824	0.920	0.271	0	1
Household socio-economic characteristics							
HHincome	Household income level: 1 = less than $10k, 2 = $10k–$15k, 3 = $15k–$25k, 4 = $25k–$35k, 5 = $35k–$50k, 6 = $50k–$75k, 7 = $75k–$100k, 8 = $100k–$125k, 9 = $125k–$150k, 10 = $150k–$200k, 11 = $200k or more	Ordinal	226,824	6.303	2.588	1	11
Hhvehcount	Number of vehicles in the household	Ordinal	226,824	2.240	1.238	0	12
Homerent	If the house is a rental	Dummy	226,824	0.211	0.408	0	1
Geographic characteristics at the home location							
Pdensity	Population density (persons per square mile) in the census block group of household’s home location in log	Continuous	226,824	7.150	1.758	3.9	10.3
Rail	If the home location has rail service	Dummy	226,824	0.157	0.364	0	1
Urban	If home is located in an urban area	Dummy	226,824	0.766	0.424	0	1
Seasons							
Spring	March, April, or May	Dummy	226,824	0.205	0.404	0	1
Summer	June, July, or August	Dummy	226,824	0.259	0.438	0	1
Fall	September, October, or November	Dummy	226,824	0.267	0.442	0	1
Winter	December, January, or February	Dummy	226,824	0.270	0.444	0	1

**Table 2:** Results for the relationship between ridesharing and public transit use (dependent variable: Rideshare).

Variables	Coef.	Std. Err.	*z*-Value	*p*-Value	Marginal Effects
non-zero state (not always 0)					
Ptused	0.012 ***	0.002	7.30	**0.000**	1.2%
Female	−0.098 ***	0.021	−4.57	**0.000**	−9.3%
Age	−0.010 ***	0.001	−12.59	**0.000**	−1.0%
Education	−0.034 **	0.012	−2.74	**0.006**	−3.4%
White	0.013	0.027	0.49	0.621	1.3%
Worker	0.008	0.029	0.29	0.772	0.8%
Driver	−0.446 ***	0.044	−10.07	**0.000**	−36.0%
HHincome	0.059 ***	0.005	12.73	**0.000**	6.0%
HHvehcount	−0.082 ***	0.009	−8.80	**0.000**	−7.9%
Homerent	0.232 ***	0.026	8.77	**0.000**	26.1%
Pdensity	0.139 ***	0.010	13.84	**0.000**	14.9%
Rail	0.110 ***	0.025	4.44	**0.000**	11.6%
Urban	−0.363 ***	0.055	−6.62	**0.000**	−30.5%
Spring	0.137 ***	0.031	4.48	**0.000**	14.7%
Summer	−0.086 **	0.029	−2.91	**0.004**	−8.2%
Winter	0.009	0.028	0.33	0.738	0.9%
Intercept	0.562 ***	0.104	5.39	0.000	
zero state (odds of always 0)					
Ptused	−0.059 ***	0.003	−18.23	**0.000**	−5.7%
Female	0.112 ***	0.023	4.93	**0.000**	11.9%
Age	0.040 ***	0.001	50.69	**0.000**	4.1%
Education	−0.464 ***	0.012	−38.42	**0.000**	−37.1%
White	−0.145 ***	0.029	−4.95	**0.000**	−13.5%
Worker	−0.361 ***	0.028	−12.97	**0.000**	−30.3%
Driver	−0.354 ***	0.050	−7.04	**0.000**	−29.8%
HHincome	−0.245 ***	0.005	−45.22	**0.000**	−21.7%
HHvehcount	0.210 ***	0.012	18.16	**0.000**	23.4%
Homerent	−0.610 ***	0.029	−20.87	**0.000**	−45.6%
Pdensity	−0.264 ***	0.011	−24.68	**0.000**	−23.2%
Rail	−0.316 ***	0.028	−11.40	**0.000**	−27.1%
Urban	−0.236 ***	0.050	−4.72	**0.000**	−21.0%
Spring	0.078 *	0.033	2.41	**0.016**	8.2%
Summer	0.120 ***	0.032	3.81	**0.000**	12.8%
Winter	−0.078 **	0.030	−2.58	**0.010**	−7.5%
Intercept	6.098 ***	0.105	57.82	0.000	
Number of obs.		226,824			
Nonzero obs.		17,030			
Zero obs.		209,794			
Log likelihood		−83,927.93			
LR chi2		1766.91 ***			

*p*-Value: * *p* < 0.05; ** *p* < 0.01; *** *p* < 0.001. (The bold figures in this table represent their *p*-Values are less than 0.05)

**Table 3:** Results for the relationship between ridesharing and public transit use varying by population density.

Variables	High Population Density (Dependent Variable: Rideshare)					Low Population Density (Dependent Variable: Rideshare)				
	Coef.	Std. Err.	*z*-Value	*p*-Value	Marginal Effects	Coef.	Std. Err.	*z*-Value	*p*-Value	Marginal Effects
	non-zero state (not always 0)					non-zero state (not always 0)				
Ptused	0.011 ***	0.002	6.44	**0.000**	1.1%	0.009 *	0.004	2.24	**0.025**	0.9%
Female	−0.080 ***	0.024	−3.29	**0.001**	−7.7%	−0.095	0.049	−1.94	0.053	−9.1%
Age	−0.011 ***	0.001	−11.43	**0.000**	−1.1%	−0.012 ***	0.002	−7.46	**0.000**	−1.2%
Education	−0.023	0.014	−1.56	0.118	−2.2%	0.056 *	0.026	2.12	**0.034**	5.8%
White	0.077 *	0.030	2.56	**0.010**	8.0%	−0.302 ***	0.069	−4.38	**0.000**	−26.1%
Worker	0.024	0.034	0.72	0.473	2.5%	0.11	0.059	1.86	0.063	11.7%
Driver	−0.460 ***	0.048	−9.63	**0.000**	−36.8%	0.213 *	0.100	2.13	**0.033**	23.7%
HHincome	0.074 ***	0.005	13.97	**0.000**	7.6%	0.060 ***	0.010	5.78	**0.000**	6.2%
HHvehcount	−0.139 ***	0.011	−12.68	**0.000**	−13.0%	−0.016	0.019	−0.83	0.407	−1.6%
Homerent	0.284 ***	0.029	9.83	**0.000**	32.8%	0.243 ***	0.067	3.61	**0.000**	27.5%
Rail	0.182 ***	0.026	7.01	**0.000**	20.0%	0.146 *	0.065	2.26	**0.024**	15.8%
Spring	0.106 **	0.035	3.00	**0.003**	11.1%	0.197 **	0.069	2.86	**0.004**	21.8%
Summer	−0.087 **	0.034	−2.60	**0.009**	−8.4%	−0.022	0.068	−0.32	0.750	−2.1%
Winter	0.018	0.032	0.56	0.575	1.8%	0.019	0.065	0.29	0.770	1.9%
Intercept	1.332 ***	0.089	14.89	0.000		−0.525 **	0.161	−3.26	0.001	
	zero state (odds of always 0)					zero state (odds of always 0)				
Ptused	−0.050 ***	0.003	−15.39	**0.000**	−4.9%	−1.794 ***	0.151	−11.91	**0.000**	−83.4%
Female	0.116 ***	0.028	4.20	**0.000**	12.3%	0.138 **	0.054	2.58	**0.010**	14.8%
Age	0.045 ***	0.001	46.39	**0.000**	4.6%	0.035 ***	0.002	19.60	**0.000**	3.6%
Education	−0.468 ***	0.015	−31.81	**0.000**	−37.4%	−0.457 ***	0.028	−16.42	**0.000**	−36.7%
White	−0.206 ***	0.034	−6.06	**0.000**	−18.6%	0.11	0.076	1.44	0.150	11.6%
Worker	−0.427 ***	0.034	−12.65	**0.000**	−34.8%	−0.285 ***	0.063	−4.52	**0.000**	−24.8%
Driver	−0.328 ***	0.056	−5.81	**0.000**	−27.9%	0.102	0.145	0.70	0.485	10.7%
HHincome	−0.232 ***	0.006	−35.83	**0.000**	−20.7%	−0.311 ***	0.013	−24.20	**0.000**	−26.7%
HHvehcount	0.254 ***	0.015	17.25	**0.000**	28.9%	0.238 ***	0.023	10.15	**0.000**	26.8%
Homerent	−0.643 ***	0.034	−18.98	**0.000**	−47.4%	−0.723 ***	0.077	−9.44	**0.000**	−51.5%
Rail	−0.489 ***	0.031	−15.77	**0.000**	−38.7%	−0.205 *	0.081	−2.52	**0.012**	−18.5%
Spring	0.091 *	0.040	2.29	**0.022**	9.5%	0.128	0.075	1.70	0.088	13.7%
Summer	0.118 **	0.038	3.10	**0.002**	12.6%	0.267 ***	0.074	3.59	**0.000**	30.7%
Winter	−0.091 *	0.037	−2.50	**0.013**	−8.7%	0.004	0.072	0.06	0.953	0.4%
Intercept	3.324 ***	0.091	36.38	0.000		3.390 ***	0.198	17.12	0.000	
Number of obs.		106,532					120,292			
Nonzero obs.		12,432					4598			
Zero obs.		94,100					115,694			
Log likelihood		−58,812.48					−24,951.1			
LR chi2		1241.85 ***					202.11 ***			

*p*-Value: * *p* < 0.05; ** *p* < 0.01; *** *p* < 0.001. (The bold figures in this table represent their *p*-Values are less than 0.05)

**Table 4:** Results for the relationship between ridesharing and public transit use varying by the household vehicle ownership.

Variables	Low Number of Household Vehicles (Dependent Variable: RIDESHARE)					High Number of Household Vehicles (Dependent Variable: Rideshare)				
	Coef.	Std. Err.	*z*-Value	*p*-Value	Marginal Effects	Coef.	Std. Err.	*z*-Value	*p*-Value	Marginal Effects
	non-zero state (not always 0)					non-zero state (not always 0)				
Ptused	0.012 ***	0.002	6.97	**0.000**	1.3%	0.005	0.004	1.45	0.147	0.5%
Female	−0.085 ***	0.024	−3.52	**0.000**	−8.2%	−0.082	0.050	−1.65	0.098	−7.9%
Age	−0.009 ***	0.001	−9.40	**0.000**	−0.9%	−0.014 ***	0.002	−7.93	**0.000**	−1.4%
Education	−0.025	0.014	−1.74	0.082	−2.5%	0.032	0.027	1.19	0.233	3.3%
White	0.034	0.030	1.13	0.259	3.5%	−0.061	0.067	−0.91	0.361	−5.9%
Worker	0.044	0.034	1.29	0.196	4.5%	0.038	0.061	0.63	0.529	3.9%
Driver	−0.511 ***	0.046	−11.19	**0.000**	−40.0%	0.176	0.127	1.39	0.165	19.2%
HHincome	0.057 ***	0.005	11.05	**0.000**	5.8%	0.070 ***	0.011	6.31	**0.000**	7.3%
Homerent	0.249 ***	0.028	8.80	**0.000**	28.3%	0.352 ***	0.078	4.49	**0.000**	42.2%
Pdensity	0.155 ***	0.011	13.70	**0.000**	16.8%	0.106 ***	0.023	4.53	**0.000**	11.2%
Rail	0.121 ***	0.028	4.35	**0.000**	12.9%	0.075	0.057	1.30	0.193	7.8%
Urban	−0.219 **	0.070	−3.14	**0.002**	−19.7%	−0.306 **	0.105	−2.93	**0.003**	−26.4%
Spring	0.121 ***	0.035	3.50	**0.000**	12.8%	0.205 **	0.072	2.85	**0.004**	22.8%
Summer	−0.062	0.033	−1.86	0.063	−6.0%	−0.142 *	0.068	−2.08	**0.037**	−13.3%
Winter	−0.001	0.031	−0.02	0.986	−0.1%	0.091	0.066	1.39	0.164	9.6%
Intercept	0.126	0.118	1.06	0.288		−0.942 ***	0.222	−4.24	0.000	
	zero state (odds of always 0)					zero state (odds of always 0)				
Ptused	−0.052 ***	0.003	−16.39	**0.000**	−5.1%	−1.615 ***	0.171	−9.47	**0.000**	−80.1%
Female	0.121 ***	0.026	4.59	**0.000**	12.9%	0.115 *	0.057	2.03	**0.043**	12.2%
Age	0.043 ***	0.001	46.39	**0.000**	4.4%	0.031 ***	0.002	15.85	**0.000**	3.1%
Education	−0.445 ***	0.014	−31.65	**0.000**	−35.9%	−0.520 ***	0.030	−17.26	**0.000**	−40.6%
White	−0.126 ***	0.033	−3.78	**0.000**	−11.8%	−0.205 **	0.078	−2.63	**0.009**	−18.6%
Worker	−0.323 ***	0.033	−9.79	**0.000**	−27.6%	−0.458 ***	0.067	−6.83	**0.000**	−36.7%
Driver	−0.248 ***	0.053	−4.68	**0.000**	−22.0%	−0.027	0.185	−0.15	0.883	−2.7%
HHincome	−0.219 ***	0.006	−36.08	**0.000**	−19.7%	−0.271 ***	0.014	−19.79	**0.000**	−23.8%
Homerent	−0.630 ***	0.032	−19.80	**0.000**	−46.7%	−0.589 ***	0.091	−6.45	**0.000**	−44.5%
Pdensity	−0.284 ***	0.012	−22.84	**0.000**	−24.8%	−0.301 ***	0.027	−11.32	**0.000**	−26.0%
Rail	−0.372 ***	0.032	−11.55	**0.000**	−31.0%	−0.235 **	0.074	−3.17	**0.002**	−21.0%
Urban	−0.185 **	0.065	−2.86	**0.004**	−16.9%	−0.327 **	0.106	−3.09	**0.002**	−27.9%
Spring	0.085 *	0.038	2.25	**0.024**	8.9%	0.081	0.081	0.99	0.322	8.4%
Summer	0.145 ***	0.037	3.97	**0.000**	15.6%	0.108	0.080	1.36	0.175	11.5%
Winter	−0.079 *	0.035	−2.26	**0.024**	−7.6%	0.004	0.076	0.05	0.960	0.4%
Intercept	6.062 ***	0.119	51.10	0.000		7.182 ***	0.278	25.85	0.000	
Number of obs.		153,158					73,666			
Nonzero obs.		12,826					4204			
Zero obs.		140,332					69,462			
Log likelihood		−62,206.96					−21,514.84			
LR chi2		1381.96 ***					183.07 ***			

*p*-Value: * *p* < 0.05; ** *p* < 0.01; *** *p* < 0.001. (The bold figures in this table represent their *p*-Values are less than 0.05)

## References

[1] United States Environmental Protection Agency (EPA) (2018). Inventory of U.S. Greenhouse Gas Emissions and Sinks. https://www.epa.gov/ghgemissions/inventory-us-greenhouse-gas-emissions-and-sinks.

[2] Adie Tomer (2017). America’s Commuting Choices: 5 Major Takeaways from 2016 Census Data. https://www.brookings.edu/blog/the-avenue/2017/10/03/americans-commuting-choices-5-major-takeaways-from-2016-census-data/.

[3] Nielsen J.R., Hovmøller H., Blyth P.L., Sovacool B.K. (2015). Of “white crows” and “cash savers:” A qualitative study of travel behavior and perceptions of ridesharing in Denmark. Transp. Res. Part A Policy Pract..

[4] Erdoğan S., Cirillo C., Tremblay J.M. (2015). Ridesharing as a green commute alternative: A campus case study. Int. J. Sustain. Trans..

[5] Chan N.D., Shaheen S.A. (2012). Ridesharing in North America: Past, present, and future. Trans. Rev..

[6] Yu B., Ma Y., Xue M., Tang B., Wang B., Yan J., Wei Y.M. (2017). Environmental benefits from ridesharing: A case of Beijing. Appl. Energy.

[7] Fagnant D.J., Kockelman K.M. (2014). The travel and environmental implications of shared autonomous vehicles, using agent-based model scenarios. Transp. Res. C Emerg. Technol..

[8] Li Z., Hong Y., Zhang Z. (2016). Do ride-sharing services affect traffic congestion? An empirical study of uber entry. Soc. Sci. Res. Netw..

[9] Jacobson S.H., King D.M. (2009). Fuel saving and ridesharing in the US: Motivations, limitations, and opportunities. Transp. Res. Part D Transp. Environ..

[10] Furuhata M., Dessouky M., Ordóñez F., Brunet M.E., Wang X., Koenig S. (2013). Ridesharing: The state-of-the-art and future directions. Transp. Res. Part B Methodol..

[11] Beirão G., Cabral J.S. (2007). Understanding attitudes towards public transport and private car: A qualitative study. Transp. Policy.

[12] (2016). Kyle Shelton. Can Public Transit and Ride-Share Companies Get Along?. http://theconversation.com/can-public-transit-and-ride-share-companies-get-along-64269.

[13] Bian Z., Liu X. A Detour-Based Pricing Mechanism for First-Mile Ridesharing in Connection With Rail Public Transit. Proceedings of the 2018 Joint Rail Conference (American Society of Mechanical Engineers).

[14] Murray G. (2012). Ridesharing as a Complement to Transit. Transportation Research Board.

[15] Babar Y., Burtch G. (2017). Examining the Impact of Ridehailing Services on Public Transit Use, SSRN Working Paper. https://ssrn.com/abstract=3042805.

[16] Stiglic M., Agatz N., Savelsbergh M., Gradisar M. (2018). Enhancing urban mobility: Integrating ride-sharing and public transit. Comput. Oper. Res..

[17] Rayle L., Shaheen S., Chan N., Dai D., Cervero R. (2014). App-Based, On-Demand Ride Services: Comparing Taxi and Ridesourcing Trips and User Characteristics in San Francisco.

[18] Masoud N., Jayakrishnan R. (2017). A real-time algorithm to solve the peer-to-peer ride-matching problem in a flexible ridesharing system. Transp. Res. Part B Methodol..

[19] Pelzer D., Xiao J., Zehe D., Lees M.H., Knoll A.C., Aydt H. (2015). A partition-based match making algorithm for dynamic ridesharing. IEEE Trans. Intell. Transp. Syst..

[20] Zhang J., Wen D., Zeng S. (2016). A discounted trade reduction mechanism for dynamic ridesharing pricing. IEEE Transactions Intell. Transp. Syst..

[21] Liu Y., Li Y. (2017). Pricing scheme design of ridesharing program in morning commute problem. Transp. Res. C Emerg. Technol..

[22] Sánchez D., Martínez S., Domingo-Ferrer J. (2016). Co-utile P2P ridesharing via decentralization and reputation management. Transp. Res. C Emerg. Technol..

[23] Li Y., Chen R., Chen L., Xu J. (2017). Towards social-aware ridesharing group query services. IEEE Trans. Serv. Comput..

[24] Burtch G., Carnahan S., Greenwood B.N. (2018). Can you gig it? An empirical examination of the gig economy and entrepreneurial activity. Manag. Sci..

[25] Caulfield B. (2009). Estimating the environmental benefits of ride-sharing: A case study of Dublin. Transp. Res. Part D: Transp. Environ..

[26] Yin B., Liu L., Coulombel N., Viguie V. (2018). Appraising the environmental benefits of ride-sharing: The Paris region case study. J. Clean. Prod..

[27] Ding C., Wang D., Liu C., Zhang Y., Yang J. (2017). Exploring the influence of built environment on travel mode choice considering the mediating effects of car ownership and travel distance. Transp. Res. Part A Policy Pract..

[28] Schneider R.J. (2013). Theory of routine mode choice decisions: An operational framework to increase sustainable transportation. Transp. Policy.

[29] Liu Y., Sheng H., Mundorf N., Redding C., Ye Y. (2017). Integrating norm activation model and theory of planned behavior to understand sustainable transport behavior: Evidence from China. Int. J. Environ. Res. Public Health.

[30] Khan M., Kockelman K.M., Xiong X. (2014). Models for anticipating non-motorized travel choices, and the role of the built environment. Transp. Policy.

[31] Efthymiou D., Antoniou C., Waddell P. (2013). Factors affecting the adoption of vehicle sharing systems by young drivers. Transp. Policy.

[32] Zolnik E.J. (2015). The effect of gasoline prices on ridesharing. J. Transp. Geogr..

[33] Dias F.F., Lavieri P.S., Garikapati V.M., Astroza S., Pendyala R.M., Bhat C.R. (2017). A behavioral choice model of the use of car-sharing and ride-sourcing services. Transportation.

[34] Rayle L., Dai D., Chan N., Cervero R., Shaheen S. (2016). Just a better taxi? A survey-based comparison of taxis, transit, and ridesourcing services in San Francisco. Transp. Policy.

[35] U.S. Department of Transportation, Federal Highway Administration 2017 National Household Travel Survey. http://nhts.ornl.gov.

[36] Lord D., Mannering F. (2010). The statistical analysis of crash-frequency data: A review and assessment of methodological alternatives. Transp. Res. Part A Policy Pract..

[37] Cameron A.C., Trivedi P.K. (2013). Regression Analysis of Count Data.

[38] Ver Hoef J.M., Boveng P.L. (2007). Quasi-Poisson vs. negative binomial regression: How should we model overdispersed count data?. Ecology.

[39] uur A.F., Ieno E.N., Walker N.J., Saveliev A.A., Smith G.M. (2009). Zero-truncated and zero-inflated models for count data. Mixed effects models and extensions in ecology with R.

[40] Fang R., Wagner B.D., Harris J.K., Fillon S.A. (2016). Zero-inflated negative binomial mixed model: An application to two microbial organisms important in oesophagitis. Epidemiol. Infect..

[41] Dong C., Clarke D.B., Yan X., Khattak A., Huang B. (2014). Multivariate random-parameters zero-inflated negative binomial regression model: An application to estimate crash frequencies at intersections. Accid. Anal. Prev..

[42] Shen S., Neyens D.M. (2017). Factors affecting teen drivers' crash-related length of stay in the hospital. J. Transp. Health.

[43] Greene W.H. (1994). Accounting for Excess Zeros and Sample Selection in Poisson and Negative Binomial Regression Models.

[44] Zhu M., Liu X.Y., Wang X. (2018). An Online Ride-Sharing Path-Planning Strategy for Public Vehicle Systems. IEEE Trans. Intell. Transp. Syst..

[45] Taylor B.D., Morris E.A. (2015). Public transportation objectives and rider demographics: Are transit’s priorities poor public policy?. Transportation.

[46] Van Lierop D., Badami M.G., El-Geneidy A.M. (2018). What influences satisfaction and loyalty in public transport? A review of the literature. Transp. Rev..

[47] Chakrabarti S. (2017). How can public transit get people out of their cars? An analysis of transit mode choice for commute trips in Los Angeles. Transp. Policy.

[48] Masoud N., Lloret-Batlle R., Jayakrishnan R. (2017). Using bilateral trading to increase ridership and user permanence in ridesharing systems. Transp. Res. Part E Logist. Transp. Rev..

